# Hemifacial Pain and Hemisensory Disturbance Referred from Occipital Neuralgia Caused by Pathological Vascular Contact of the Greater Occipital Nerve

**DOI:** 10.1155/2017/3827369

**Published:** 2017-02-26

**Authors:** Byung-chul Son, Jin-gyu Choi

**Affiliations:** ^1^Department of Neurosurgery, Seoul St. Mary's Hospital College of Medicine, The Catholic University of Korea, Seoul, Republic of Korea; ^2^Catholic Neuroscience Institute, College of Medicine, The Catholic University of Korea, Seoul, Republic of Korea

## Abstract

Here we report a unique case of chronic occipital neuralgia caused by pathological vascular contact of the left greater occipital nerve. After 12 months of left-sided, unremitting occipital neuralgia, a hypesthesia and facial pain developed in the left hemiface. The decompression of the left greater occipital nerve from pathological contacts with the occipital artery resulted in immediate relief for hemifacial sensory change and facial pain, as well as chronic occipital neuralgia. Although referral of pain from the stimulation of occipital and cervical structures innervated by upper cervical nerves to the frontal head of V1 trigeminal distribution has been reported, the development of hemifacial sensory change associated with referred trigeminal pain from chronic occipital neuralgia is extremely rare. Chronic continuous and strong afferent input of occipital neuralgia caused by pathological vascular contact with the greater occipital nerve seemed to be associated with sensitization and hypersensitivity of the second-order neurons in the trigeminocervical complex, a population of neurons in the C2 dorsal horn characterized by receiving convergent input from dural and cervical structures.

## 1. Introduction

It is well known that patients with primary headaches often report pain not only involving the frontal head innervated by the first (ophthalmic) division of the trigeminal nerve, but also involving the occipital region innervated by the greater occipital nerve (GON), a branch of the C2 spinal root [1–3]. Likewise, stimulation of structures in the neck innervated by upper cervical roots in humans such as infratentorial dura mater, vessels, and tumors of the posterior fossa can lead to pain in the front of the head [4–7]. Trigeminocervical complex (TCC) is a population of neurons in the C2 dorsal horn characterized by receiving convergent input from dural and cervical structures [3]. These neurons show properties typical of dura-sensitive trigeminal neurons with a convergent input from the facial skin corresponding to the dermatome of the ophthalmic division of the trigeminal nerve [3, 8, 9]. This convergence of nociceptive afferents and sensitization of TCC neurons have clinical correlates such as hypersensitivity and the spread and referral of pain frequently seen in patients with primary headaches such as migraines.

Clinical evidence of referred pain in the frontal head with involvement of the TCC from cervical lesions has been reported in cases of posterior fossa tumors, stimulation of the infratentorial dura mater, direct stimulation of cervical roots, vertebral artery dissection, and stimulation of subcutaneous tissue innervated by the GON [3, 9]. However, the development of hemifacial sensory change associated with referred trigeminal pain from occipital neuralgia is extremely rare. We report a case of hemifacial sensory change associated with referred trigeminal pain from chronic occipital neuralgia. Furthermore, occipital neuralgia was found to be caused by pathological vascular contact with the GON. Decompression of the GON immediately resulted in the disappearance of hemisensory deficit and hemifacial pain.

## 2. Case Presentation

A 53-year-old female with a 16-month history of chronic stabbing pain along the distribution of the left GON presented with a left-sided facial pain with 4 months of duration. A severe aching pain and tenderness over the left occipital area were associated during initial development of left occipital pain 16 months ago. There was no precipitating event before the onset of left occipital neuralgia. The onset was described as rather sudden. The pain was described as mainly stabbing and electric in nature with moderate severity (4–6/10 on numerical rating scale (NRS) ranging from 0 to 10). It was not aggravated with neck motion. Daily activity did not influence the continuous occipital pain. Her medical history was unremarkable, including diabetes and gout. Under the impression of occipital neuralgia, she had been treated with several nonsteroidal anti-inflammatory drugs, including tramadol (150 mg/day), carbamazepine (400 mg/day), and pregabalin (225 mg/day), with some degree of relief. Pain was so agonizing that treatment of repeated occipital nerve blocks on a regular schedule of a month for a year was given. The occipital nerve block over the tender point along the course of the GON was quite effective for the initial two injections. However, its effect lasted 3 hours thereafter.

Four months prior to presentation, an aching pain developed insidiously in the left orbit, cheek, temple, and left ear canal. Subsequently, paresthesia and numbness progressively developed in her left hemiface. Despite the development of left hemifacial pain and sensory disturbance, left occipital neuralgia was not aggravated. It remained present as it had been for the prior 16 months. Considering the chronic nature of left occipital neuralgia with superimposed occurrence of hemifacial pain and sensory changes, she was referred to us for further evaluation of the trigeminal and occipital pain.

**Figure 1:**
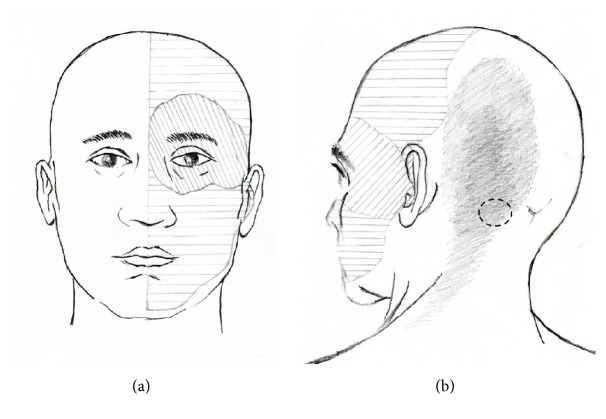
A schematic diagram demonstrating the distribution of occipital neuralgia and hemifacial sensory change with facial pain. (a) The grey areas over the left occipital area indicate the distribution of stabbing pain of occipital neuralgia. A tender point was present along the course of the greater occipital nerve. (b) The obliquely hatched area denotes regions of facial pain in addition to hypesthesia. The horizontally hatched area shows the distribution of hemifacial hypesthesia and paresthesia.

Upon examination, her left occipital pain was typically that of occipital neuralgia fulfilling the criteria of International Classification of Headache Disorders (ICHD) [10]. The stabbing occipital pain was present throughout the day with intermittent aggravation. It was present over the distribution of the left GON (Figure 1(a)). The tenderness which was severe at the onset was mild in our examination. Hemifacial pain was most severe in the left periorbital and temple. It was aching in nature. A moderate degree of hypesthesia to light touch, increased sensitivity to pinprick, and paresthesia were observed in her right face (V1, V2, and V3) (Figure 1(b)). However, no pain or hypesthesia was observed in the intraoral structures, including the buccal mucosa and the tongue. Corneal sensation was preserved. No allodynia was detected in the hypoesthetic left face. There was no tinnitus, visual disturbance, or lacrimation associated with the hemifacial pain. Repeated blocks with 1% lidocaine (3 mL) over the occipital tender point were effective for approximately 2 hours in relieving occipital neuralgia. However, the left-sided hemifacial pain and sensory disturbance were not influenced by the block.

**Figure 2:**
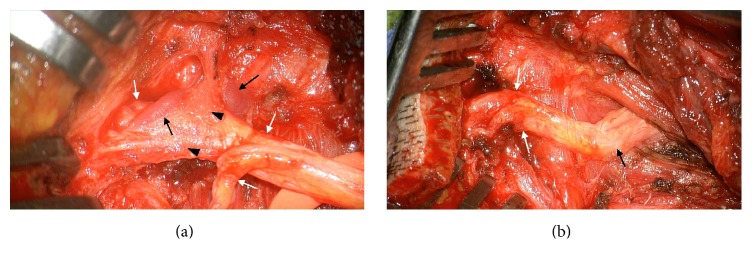
Intraoperative photographs during the decompression of the greater occipital nerve. (a) An intraoperative photograph showing adhesions of the greater occipital nerve* (white arrows)* with the connective tissue* (black arrow heads)* and occipital arteries* (black arrows)*. (b) An intraoperative photograph after decompression of the left greater occipital nerve. A reddish deformation is apparent in the compressed portion* (white arrows) *with pathological vascular contact compared to the normal proximal course* (black arrow)* of the greater occipital nerve.

Laboratory examinations were normal, including erythrocyte sedimentation rate and C-reactive protein, antinuclear antibody, and anti-DS-DNA. Cerebrospinal fluid examination was also normal. Pathological lesion to explain the left occipital and trigeminal pain was not observed in the enhanced MRI of the brain and cervical spine. Considering chronicity and medical intractability, exploration of the left GON was proposed. An approximately 8 cm sized hockey stick shaped incision and subcutaneous flap were elevated from the midline to identify the course of the GON. No entrapment of the GON was found in its course in the trapezius or semispinalis capitis. Upon dissection along the distal course of the GON, a severe adhesion to the surrounding connective tissue and entrapment of the GON between tributaries of the occipital artery were encountered (Figure 2(a)). The occipital artery and veins were dissected and cut. The connective tissue adhesion around the GON was released to decompress the GON (Figure 2(b)). Immediately after the decompression of the GON, chronic stabbing pain of the occipital neuralgia and the left hemifacial pain as well as a sensory disturbance completely disappeared. Hypesthesia and paresthesia, aching hemifacial pain, and occipital neuralgia did not recur at 12 months of follow-up.

## 3. Discussion

### 3.1. Clinical Examples of V1 Trigeminal Pain Referred from Cervical Pathology

We present a rare case of neurological deficit in addition to typical referred pain to ipsilateral V1 trigeminal pain from chronic occipital neuralgia. It is extremely difficult to find a case in the literature describing the development of hemifacial hypesthesia associated with referred facial pain from cervical pathology. Of particular interest with regard to our case is the occurrence of cluster headaches, a rare form of primary headache marked by unilateral excruciating pain in association with autonomic features, associated with an ipsilateral vertebral artery aneurysm [11] and vertebral artery dissection [12]. The secondary cluster headache from a vertebral artery aneurysm was resolved when the aneurysm was clipped [11] or treated with sumatriptan (6 mg subcutaneously) [12]. These findings provided clinical affirmation of the existence of trigeminal/cervical convergence and hypersensitivity.

### 3.2. Trigeminocervical Complex as a Substrate of Convergence and Hypersensitivity

The upper cervical spinal roots can contribute to sensory innervation of cranial and cervical structures. Occipital and suboccipital structures such as vessels, the dura mater of the posterior fossa, deep paraspinal neck muscle, and zygapophyseal joints are recognized as sources of head and neck pain [1, 9, 13]. Nociceptive inflow from these suboccipital structures is mediated by small-diameter afferent fibers in the upper cervical roots terminating from the C2 segment up to the medullary dorsal horn [9, 14, 15]. The major afferent contribution is mediated by the spinal root C2 peripherally represented by the GON [9, 16]. An anatomical overlap of the trigeminal and cervical afferents through the TCC from the level of the caudal trigeminal nucleus to at least the C2 segment has already been suggested [17]. Furthermore, an electrophysiological study [3] has described the convergence of dural afferents and cervical afferents in the GON on neurons in the TCC, with subsequent sensitization of dural input by stimulation of the GON, suggesting a neural mechanism of hypersensitivity, spread, and referral form structures of the upper cervical spine in the trigeminal domain [9].

Sensitization of the central nociceptive neurons in the TCC takes place in response to strong dural noxious inputs seen in secondary headache syndromes such as meningitis, subarachnoid hemorrhage, and experimental headaches [9]. Sensitization of these second-order neurons in the TCC could be explained by the following mechanisms: an increased afferent inflow from the periphery or central pain-modulatory influences can actively facilitate or disinhibit afferent inflow into the TCC. Occipital neuralgia in the case described herein caused by continuous vascular contact and compression could be a typical example of increased peripheral afferent inflow which resulted in the sensitization of central nociceptive neurons. The clinical correlates of this hypersensitivity include the development of spontaneous pain, hyperalgesia, and allodynia [18, 19]. In line with this, spontaneous pain and hyperalgesia to pinprick in addition to hemifacial hypesthesia were noted in the current case.

An occurrence of hemifacial hypesthesia not confined to V1 trigeminal distribution was observed in this case. This type of extension of a sensory deficit has been described in complex regional pain syndrome (CRPS) [20, 21]. Sensory deficit in patients with CRPS frequently extends past the painful area of the affected limb [20]. Increased frequency of mechanical allodynia and movement disorders in patients with hemisensory impairment or sensory deficits in the upper quadrant of the body may indicate central mechanisms. For example, functional alterations in the central processing of noxious stimuli are involved in the pathogenesis of an extension of the sensory deficit [20]. It is now well established that pain-modulating circuits in the brain stem such as the ventrolateral division of the periaqueductal grey (PAG), nucleus raphe magnus, and the rostroventral medulla are closely involved in the promotion of central sensitization and secondary hyperalgesia [10, 22]. The dynamic plasticity of descending pain-modulating pathways after peripheral nerve injury or continuous strong noxious inflow can lead to neuropathic pain and render the system vulnerable, resulting in pathological consequences [22].

### 3.3. Vascular Contact as a Cause of Occipital Neuralgia

While occipital neuralgia is mostly considered idiopathic, specific causes of occipital neuralgia should be excluded from individual cases [23–36]. The etiology in the present case is indeed the same as that reported by Cornely et al. [36], in which the GON was entrapped by pathological contact with the occipital artery. In their report, a 66-year-old woman with severe right-sided occipital neuralgia showed a severe tenderness over the trunk of the right GON with a strong pulsation of the occipital artery branch. We speculate that multiple and repeated injections of local anesthetics and steroids over the tender point during the past 16 months prior to presentation might have resulted in adhesion of connective tissues around the injection points around the pathological arterial contacts. Decompression and neurolysis with the removal of pathological arterial contacts led to immediate and complete relief of the referred hemifacial pain and chronic occipital neuralgia with immediate restoration of hemifacial sensory loss. Therefore, the contact between the GON and the occipital vessels was confirmed as the etiology of chronic occipital neuralgia with referred trigeminal pain and hemifacial sensory loss in this case.

## 4. Conclusion

An occurrence of hemifacial sensory disturbance associated with referred pain of trigeminal distribution from chronic occipital neuralgia due to pathological vascular contact of the greater occipital nerve is reported here. The present case may indicate that disturbance of sensory processing in higher central structures such as the thalamus may occur in addition to sensitization and hypersensitivity of the second-order neurons in the trigeminocervical complex, a population of neurons in C2 dorsal horn characterized by receiving convergent input from dural and cervical structures.
